# Integrated Physiological and Transcriptomic Analyses Reveal That Arbuscular Mycorrhizal Symbiosis Enhances Iron Stress Tolerance in *Eucalyptus grandis*

**DOI:** 10.3390/plants15142213

**Published:** 2026-07-20

**Authors:** Bingjie Huang, Wei Chen, Yanjing Yu, Siyuan Li, Sijia Wang

**Affiliations:** Guangxi Key Laboratory of Forest Ecology and Conservation, Guangxi Colleges and Universities Key Laboratory for Cultivation and Utilization of Subtropical Forest Plantation, School of Forestry, Guangxi University, Nanning 530004, China

**Keywords:** arbuscular mycorrhizal fungi, iron stress, *Eucalyptus grandis*, photosynthesis, antioxidant defense, MYB transcription factors

## Abstract

Iron (Fe) is an essential micronutrient for plants. However, both iron deficiency and excess can severely inhibit plant growth and productivity. Arbuscular mycorrhizal (AM) fungi have been shown to improve plant mineral nutrition and stress tolerance, yet the integrated physiological and molecular mechanisms underlying AM-mediated iron homeostasis regulation, particularly in woody tree species, remain poorly understood. In this study, we investigated the effects of inoculating *Eucalyptus grandis* seedlings with the AM fungus *Rhizophagus irregularis* on their growth, photosynthetic performance, antioxidant defense, and transcriptional responses under varying Fe supply levels (5, 25, and 200 µM). Our results demonstrated that AM symbiosis significantly alleviated the growth inhibition induced by both low-Fe (5 µM) and high-Fe (200 µM) stress, enhanced photosynthetic capacity, as evidenced by increased net photosynthetic rate (Pn), stomatal conductance (Gs), and PSII photochemical efficiency; under low-Fe, Pn, Gs, and Fv/Fm increased by 33.7%, 42.4%, and 25.2%, respectively. AM symbiosis also significantly enhanced the activities of antioxidant enzymes (POD, SOD, and CAT) under high-iron stress, accompanied by reduced accumulation of reactive oxygen species and lipid peroxidation; specifically, POD, SOD, and CAT activities increased by 79.3%, 88.7%, and 87.0%, respectively. Transcriptomic analysis identified 44 MYB transcription factors that were differentially induced by AM symbiosis under iron stress, among which six genes (*EgMYB-2*, *EgMYB-3*, *EgMYB315-1*, *EgMYB315-2*, *EgMYB61*, and *EgMYB306*) were significantly upregulated by AM under both low- and high-iron conditions, as verified by qRT-PCR. Correlation analysis revealed strong positive associations between the expression of these EgMYB genes and antioxidant enzyme activities as well as photosynthetic parameters, suggesting their potential involvement in coordinating iron stress responses. Overall, our findings indicate that AM fungus enhances iron stress tolerance in *E. grandis* through a multilevel strategy that includes photosynthetic protection, antioxidant defense activation, and transcriptional reprogramming involving MYB transcription factors as potential regulators. This study provides integrated physiological-molecular analysis and novel insights into AM-mediated Fe homeostasis regulation in the woody tree species *E. grandis*.

## 1. Introduction

Iron is an essential nutrient for plant growth [1]. For plants, iron is also an indispensable factor in various physiological and metabolic processes. Within plant cells, iron is involved in photosynthesis, mitochondrial respiration, chlorophyll biosynthesis, and the structural maintenance of numerous enzymes [2,3,4]. As a key factor in numerous redox reactions, iron participates in critical steps of the electron transport chain, directly influencing plant energy metabolism and carbon assimilation efficiency. Although iron is abundant in the Earth’s crust, its availability in soil is often low. Particularly in alkaline or calcareous soils, iron primarily exists as insoluble trivalent iron (Fe^3+^), making it difficult for plant roots to absorb effectively [5,6]. In addition to iron deficiency, iron toxicity can also harm plants by generating large amounts of reactive oxygen species (ROS), leading to membrane lipid peroxidation, protein oxidative degradation, and DNA damage [7]. Therefore, plants have to precisely regulate iron uptake, transport, storage, and utilization to maintain intracellular iron homeostasis.

Arbuscular mycorrhizal (AM) fungi belong to the phylum Glomeromycota and form mutualistic symbiotic relationships with more than 70% of the terrestrial plants [8,9]. Through their extensive extraradical hyphal networks, AM fungi assist host plants in absorbing water and mineral nutrients (particularly phosphorus), while obtaining photosynthetic products from the plants to complete their own life cycle [10,11]. Numerous studies have shown that AM symbiosis can significantly improve the status of plant nutrients such as phosphorus, and zinc, and enhance plant adaptability under abiotic stress conditions such as heavy metal stress, drought stress, and salt stress [12,13,14]. Regarding iron nutrition, AM symbiosis has been shown to boost the Fe level in certain plant species under conditions of low Fe availability [15,16]. Recent studies have revealed that AM symbiosis modulates host gene expression related to iron homeostasis at the transcriptional level, with changes in transporter and transcription factor genes detectable during symbiosis establishment [17]. In sunflowers (*Helianthus annuus*), AM fungi inoculation significantly increased plant iron content under iron-deficient conditions and upregulated the expression of iron transporter genes [18]. However, the regulatory effects of AM fungi on iron stress are clearly host-dependent [19], and the mechanisms by which AM symbiosis affects forest tree species under different iron supply levels remain unclear.

When plants are subjected to iron stress, the balance between the production and elimination of intracellular ROS is disrupted, leading to oxidative stress [20]. Plants eliminate excess ROS by activating enzymatic and non-enzymatic antioxidant systems, among which peroxidase (POD), superoxide dismutase (SOD), and catalase (CAT) are key antioxidant enzymes [21,22]. Therefore, photosynthetic parameters and chlorophyll fluorescence parameters are often used as important physiological indicators to assess the severity of iron stress and the alleviating effects of AM fungi.

MYB transcription factors constitute one of the largest transcription factor families in plants and are widely involved in secondary metabolism, cell differentiation, hormone signaling, and responses to biotic and abiotic stresses [23]. The genome-wide identification and classification of the MYB gene family has been investigated in numerous plant species, including *Arabidopsis thaliana*, poplar, apple (*Malus domestica*), banana (*Musa acuminata*), *Vitis vinifera*, and sweet cherry (*Prunus avium* L.) [24,25,26,27]. *AtMYB10* and *AtMYB72* have been shown to regulate the expression of iron absorption-related genes in the root under iron-deficient conditions [28], and the involvement of MYB transcription factors in iron homeostasis has been extensively reviewed [29]. And MYB30 is significantly upregulated under iron-deficient conditions in *Arabidopsis* [30]. In woody species, studies on MYB transcription factors in response to iron stress are still limited but emerging. In *Malus halliana*, 29 R2R3-MYB transcription factors were identified under Fe deficiency stress, with MhR2R3-MYB4 playing an important role in Fe deficiency responses [31]. In *Malus xiaojinensis*, *MxMYB1* was upregulated by Fe starvation in roots, suggesting a potential role in iron nutrition [32]. In poplar, genome-wide analysis identified 152 MYB-related transcription factors with diverse expression patterns under stress conditions, implying their crucial roles in stress responses including antioxidant defense regulation [33]. However, systematic studies on the role of MYB transcription factors in AM fungal regulation of iron stress adaptation in forest trees are still lacking.

*Eucalyptus grandis* is one of the most widely planted fast-growing timber tree species in the world, possessing excellent characteristics such as rapid growth, strong adaptability, and high biomass [34]. *E. grandis* is of great value for timber production and ecological restoration; however, it often exhibits stunted growth during the seedling and young stand stages due to insufficient or excessive soil iron supply. Furthermore, as a highly mycorrhiza-dependent species, preliminary studies have examined the symbiotic relationship between *E. grandis* and AM fungi; however, there are no reports on how AM symbiosis regulates the growth, photosynthetic performance, and antioxidant systems of *E. grandis* under different iron supply levels, nor on whether MYB transcription factors are involved in these processes. To investigate these questions, we designed a controlled experiment with different Fe supply levels based on previously reported soil available Fe concentrations in *Eucalyptus* plantations [and concentrations used in woody plant iron nutrition studies [35,36]. We hypothesized that AM symbiosis alleviates Fe deficiency and toxicity through physiological strategies (enhancing photosynthesis under low-Fe and activating antioxidant defense under high-Fe), with MYB transcription factors potentially involved in these processes. 

To address this scientific gap, we used *E. grandis* as the subject and established three iron supply levels (low iron: 5 µM; control: 25 µM; high iron: 200 µM). By inoculating or not inoculating the roots with *Rhizophagus irregularis*, the study systematically investigated the effects of AM symbiosis on the iron stress adaptation of *E. grandis*. Specific research objectives include: (1) evaluating the effects of AM fungi on the growth parameters and mycorrhizal infection rates of *E. grandis* under different iron concentrations; (2) analyzing the regulatory role of AM fungi on the photosynthetic parameters and chlorophyll fluorescence characteristics of *E. grandis* under different iron supply conditions; (3) measuring antioxidant enzyme activity to assess the mitigating effects of AM fungi on iron-stress-induced oxidative damage; (4) to identify and validate the expression profiles of MYB transcription factors regulated by AM fungi and iron stress based on transcriptomic data. This study aims to elucidate the mechanisms by which AM fungi enhance the tolerance of *E. grandis* to iron stress at both physiological and molecular levels, thereby advancing our understanding of plant-microbe interactions under nutrient stress and providing a theoretical basis for the cultivation and application of mycorrhizal *E. grandis* seedlings under iron-stressed site conditions.

## 2. Results

### 2.1. AM Fungal Colonization Promotes the Growth of Eucalyptus grandis

**Figure 1:**
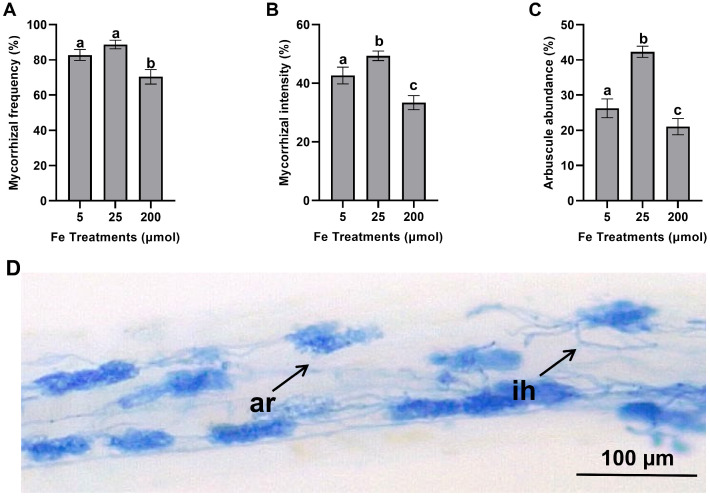
Arbuscular mycorrhizal colonization in *Eucalyptus grandis* inoculated with *Rhizophagus irregularis* under low (5 µM), control (25 µM), and high (200 µM) iron concentrations. (**A**) Mycorrhizal frequency (%), (**B**) mycorrhizal intensity (%) and (**C**) arbuscule abundance (%). Different letters indicate significant differences at *p* < 0.05 (one-way ANOVA, Tukey’s test). Data are means ± SE (*n* = 3). (**D**): Microscopic structures of AM fungal colonization in roots. Roots were stained with trypan blue. AM fungal structures including intraradical hyphae (ih) and arbuscules (ar) are indicated by arrows. Scale bar = 100 μm.

Under different iron concentration treatments, the root systems of E. grandis inoculated with R. irregularis developed typical arbuscular mycorrhizal structures, including intraradical hyphae, arbuscules, and vesicles. No fungal structures were observed in the root systems of non-mycorrhizal (NM) plants. Mycorrhizal colonization parameters are shown in Figure 1. The mycorrhizal frequency (F%) did not differ significantly between the 5 µM and 25 µM treatments, but both were significantly higher than that of the 200 µM treatment (Figure 1A). Infection intensity (M%) and arbuscule abundance (A%) were highest in the 25 µM treatment (control), significantly higher than in the 5 µM (low iron) and 200 µM (high iron) treatments; simultaneously, infection intensity and arbuscule abundance in the 5 µM treatment were also significantly higher than in the 200 µM treatment (Figure 1B,C). Representative microscopic images of AM colonization are shown in Figure 1D. These results indicate that high iron stress may significantly inhibit the colonization frequency, mycorrhizal intensity, and arbuscule development of AM fungi, whereas low iron stress primarily affects the mycorrhizal intensity and arbuscule formation within the root system.

**Figure 2:**
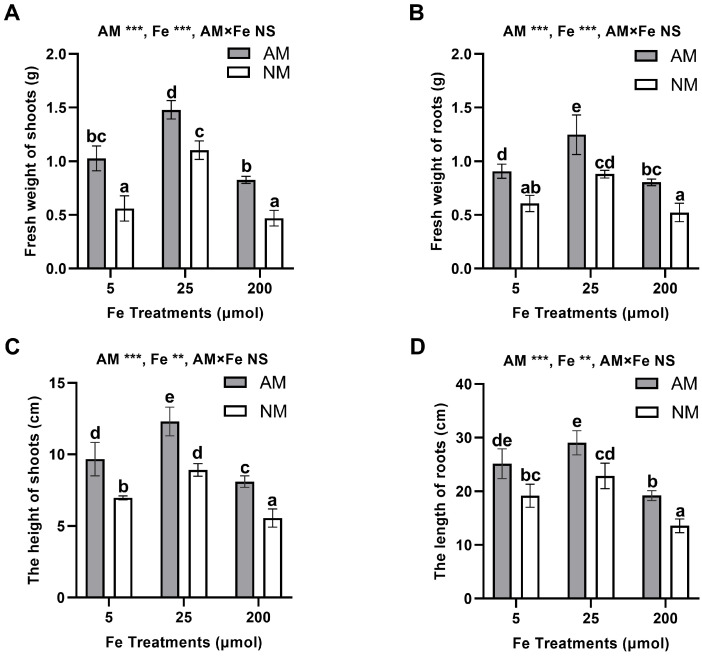
Effects of AM inoculation and iron supply on growth parameters of *E. grandis*. (**A**) Shoot fresh weight, (**B**) root fresh weight, (**C**) plant height, (**D**) root length. NM, non-mycorrhizal; AM, mycorrhizal; Low Fe (5 µM), Control Fe (25 µM), High Fe (200 µM). Different letters indicate significant differences (two-way ANOVA, Tukey’s test, *p* < 0.05). Data are means ± SE (*n* = 3). Significant effect: **: *p*  <  0.01, ***: *p * <  0.001, NS: no significant effect.

Iron concentration and AM inoculation significantly affected the growth of *E. grandis*. Under 25 µM iron concentration, the shoots and root fresh weights of AM plants were significantly higher than those of NM plants (Figure 2A,B). Low iron stress caused a significant decrease in plant height and root length of NM plants, while AM inoculation significantly alleviated this inhibitory effect (Figure 2C,D). High iron stress also inhibited growth; however, the shoots and roots fresh weight, plant height, and root length of AM-inoculated plants were all significantly higher than those of the corresponding NM plants (Figure 2). Meanwhile, NM plants exhibited poorer leaf phenotypes under iron stress, with more pronounced leaf yellowing compared to AM-inoculated plants. The root to shoot biomass ratio was also calculated to characterize the adaptive response to iron stress. Under low- and high-iron stress, the root/shoot ratio of NM plants increased significantly compared to the control, indicating that plants allocated more biomass to roots under stress conditions (Supplementary Figure S1). In contrast, AM inoculation significantly reduced the root to shoot ratio compared to NM plants under both stress conditions, suggesting that AM symbiosis alleviated the stress-induced biomass allocation shift and restored the balance between shoot and root growth.

### 2.2. AM Fungi Modulate Iron Accumulation in Eucalyptus grandis Under Iron Stress

**Figure 3:**
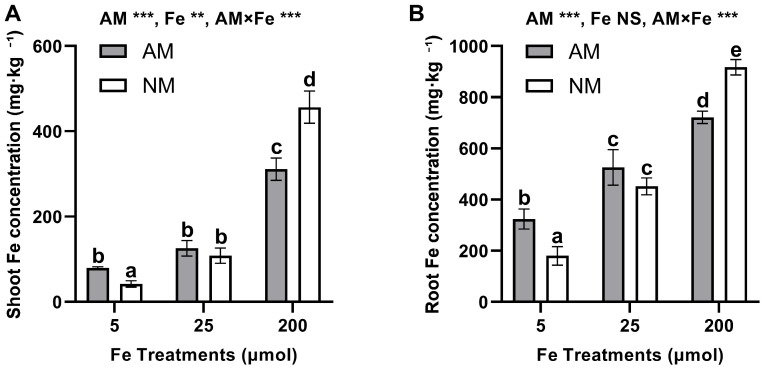
Iron concentrations in shoots (**A**) and roots (**B**) of *E. grandis* under different iron supply levels (5, 25, and 200 µM) with or without AM inoculation. Different letters indicate significant differences (two-way ANOVA, Tukey’s test, *p* < 0.05). Data are means ± SE (*n* = 3). Significant effect: **: *p*  <  0.01, ***: *p * <  0.001, NS: no significant effect.

Under low iron conditions, AM inoculation significantly increased shoot Fe concentration by 91.5% (from 41.55 to 79.58 mg·kg^−1^) and root Fe concentration by 79.8% (from 180.08 to 323.73 mg·kg^−1^) compared to NM plants (Figure 3A,B). Under control conditions, there were no significant differences in Fe concentration between AM and NM plants in either shoots or roots. Under high iron conditions, AM inoculation significantly reduced shoot Fe concentration by 31.9% (from 456.47 to 311.07 mg·kg^−1^) and root Fe concentration by 21.4% (from 917.29 to 721.32 mg·kg^−1^) compared to NM plants. These results indicate that AM symbiosis is associated with differential regulation of iron uptake and distribution in *E. grandis*, promoting Fe acquisition under deficiency while preventing excessive accumulation under toxicity.

### 2.3. Effects of AM Fungi on Photosynthetic Performance of Eucalyptus grandis Under Iron Stress

**Figure 4:**
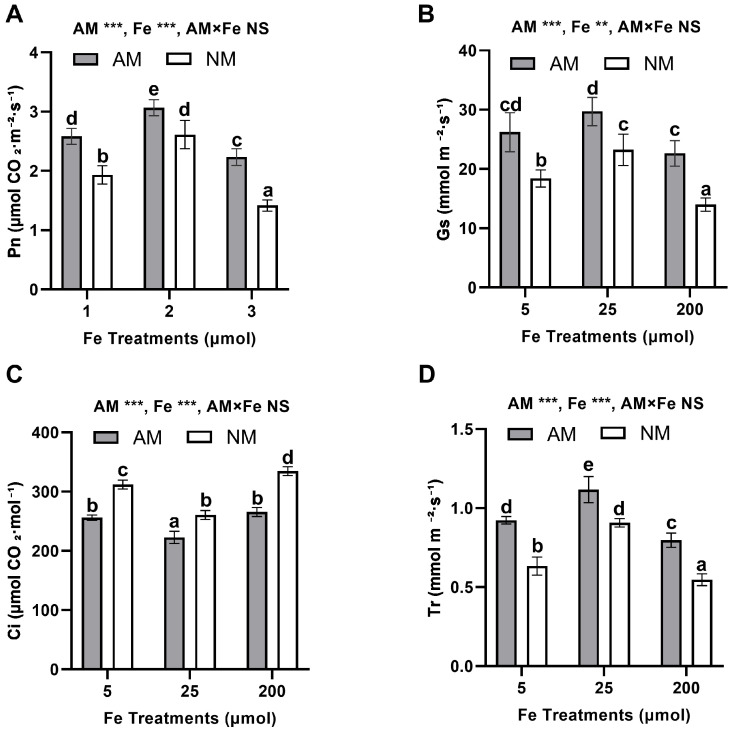
Effects of AM inoculation and iron stress on leaves photosynthetic parameters of *E. grandis*. (**A**) Net photosynthetic rate (Pn), (**B**) stomatal conductance (Gs), (**C**) intercellular CO_2_ concentration (Ci), (**D**) transpiration rate (Tr). Different letters indicate significant differences (two-way ANOVA, Tukey’s test, *p* < 0.05). Data are means ± SE (*n* = 3). Significant effect: **: *p*  <  0.01, ***: *p*  <  0.001, NS: no significant effect.

Photosynthesis is a key physiological indicator for assessing a plant’s response to environmental stress. To determine the effects of AM symbiosis on the photosynthetic performance of *E. grandis* leaves under iron stress, we measured gas exchange parameters under different treatment conditions. Under control iron concentration (25 µM), the net photosynthetic rate (Pn) of AM plants was significantly higher than that of NM plants. Low iron stress (5 µM) caused a significant decrease in Pn of NM plants, while Pn of AM plants remained at the control level, significantly higher than that of NM plants. Under high iron stress (200 µM), the decline in Pn was even more pronounced in NM plants; although Pn decreased in AM plants, it remained significantly higher than that of NM plants (Figure 4A). Stomatal conductance (Gs) and transpiration rate (Tr) exhibited similar trends (Figure 4B,D), with AM inoculation significantly mitigating the declines in Gs and Tr caused by iron stress. Intercellular CO_2_ concentration (Ci) showed an upward trend under both low- and high-iron conditions, but Ci in AM-inoculated plants was significantly lower than in NM-inoculated plants (Figure 4C). These results indicate that AM fungi effectively alleviated the inhibitory effects of iron stress on the photosynthesis of *E. grandis* by maintaining higher photosynthetic capacity.

### 2.4. AM Fungi Alleviate the Inhibitory Effects of Iron Stress on Chlorophyll Fluorescence in Eucalyptus grandis

**Figure 5:**
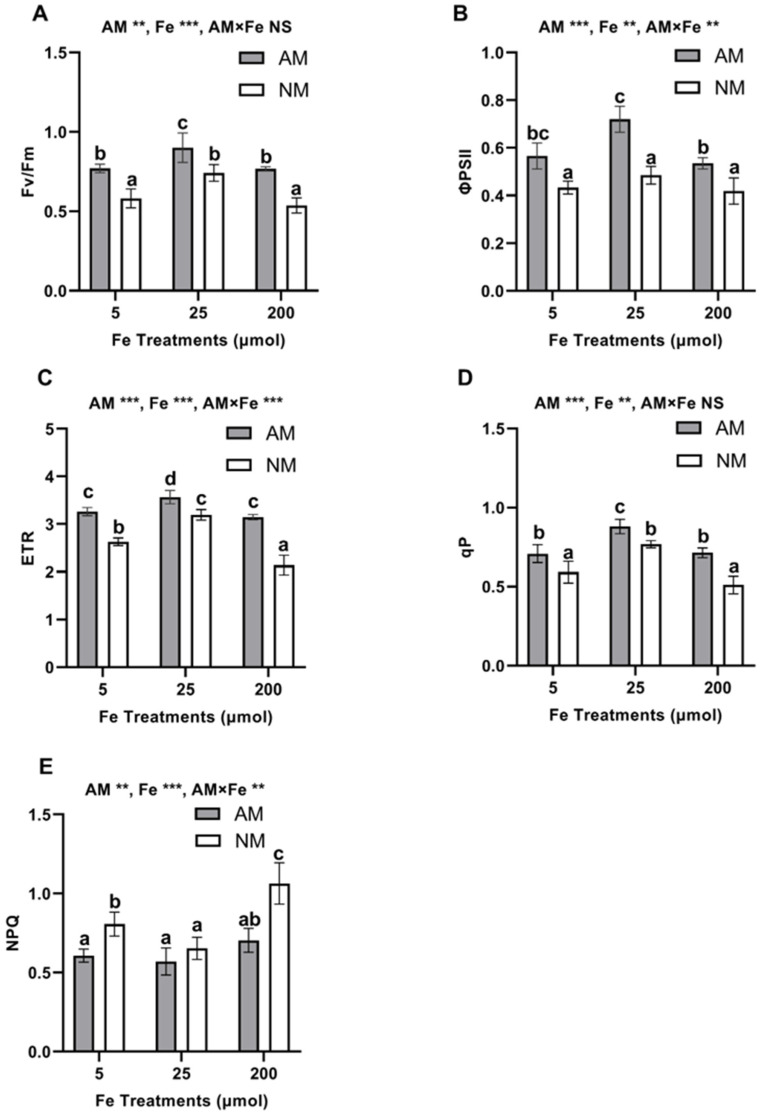
Effects of AM inoculation and iron stress on chlorophyll fluorescence parameters in leaves of *E. grandis*. (**A**) Maximum quantum efficiency of PSII (Fv/Fm), (**B**) actual photochemical efficiency of PSII (ΦPSII), (**C**) electron transport rate (ETR), (**D**) photochemical quenching (qP), (**E**) non-photochemical quenching (NPQ). Different letters indicate significant differences (two-way ANOVA, Tukey’s test, *p*  <  0.05). Data are means ± SE (*n* = 3). Significant effect: **: *p*  <  0.01, ***: *p*  <  0.001, NS: no significant effect.

To investigate the effects of AM fungi on the photochemical efficiency of photosystem II (PSII) in *E. grandis* under iron stress, we analyzed chlorophyll fluorescence parameters. At the control iron concentration, the maximum photochemical quantum yield (Fv/Fm) of AM plants was higher than that of NM plants. Under low- and high-iron stress conditions, Fv/Fm in NM plants decreased significantly, whereas Fv/Fm in AM plants remained at a higher level, significantly higher than that of NM plants (Figure 5A). Under all three iron concentration treatments, the actual photochemical efficiency (ΦPSII) and electron transfer rate (ETR) of PSII in AM plants were both significantly higher than those in NM plants (Figure 5B,C). Under both low-iron and high-iron conditions, the photochemical quenching coefficient (qP) of AM plants was higher than that of NM plants, while non-photochemical quenching (NPQ) was significantly lower than that of NM plants (Figure 5D,E). These results indicate that AM fungi can mitigate damage to the PSII reaction center caused by iron stress, maintain high conversion efficiency and electron transport rates, suggesting a potential association with the preservation of functional integrity of the photosynthetic electron transport chain.

### 2.5. Effects of AM Fungi on Reactive Oxygen Species Accumulation and Lipid Peroxidation in Eucalyptus grandis Under Iron Stress

**Figure 6:**
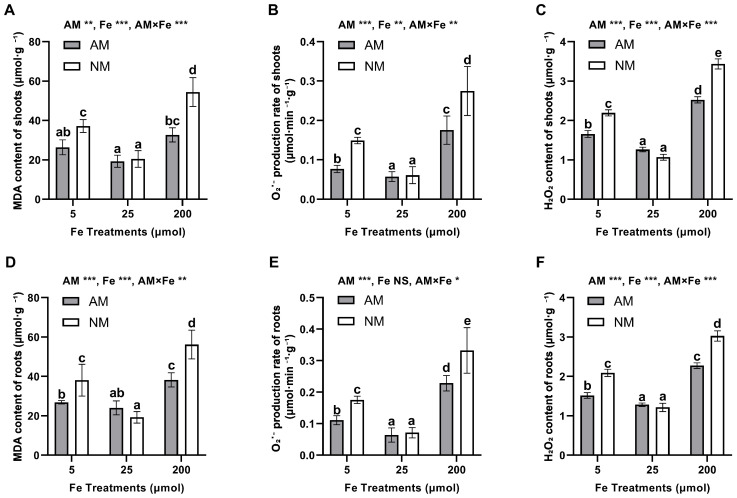
Effects of AM inoculation on oxidative stress markers in shoots and roots of *E. grandis* under different iron supply levels (5, 25, and 200 µM). Malondialdehyde (MDA) content of shoots (**A**) and roots (**D**); Superoxide anion (O_2_^•−^) production rate of shoots (**B**) and roots (**E**); Hydrogen peroxide (H_2_O_2_) content of shoots (**C**) and roots (**F**). Different lowercase letters indicate significant differences among treatments (Tukey’s test, *p* < 0.05). Significant effect: *: *p*  <  0.05, **: *p*  <  0.01, ***: *p*  <  0.001, NS: no significant effect.

To directly assess whether AM symbiosis alleviates oxidative stress in *E. grandis* under iron stress, we measured malondialdehyde (MDA) content, superoxide anion (O_2_^•−^) production rate, and hydrogen peroxide (H_2_O_2_) content in shoots and roots under different treatments. Under control iron concentration (25 µM), there were no significant differences in MDA, O_2_^•−^, or H_2_O_2_ levels between AM and NM plants (Figure 6). Low-iron stress (5 µM) induced moderate increases in these oxidative stress markers in NM plants, while AM inoculation significantly reduced their accumulation in both tissues. High-iron stress (200 µM) caused the most severe increases in NM plants, with MDA content, O_2_^•−^ production rate, and H_2_O_2_ content increasing dramatically. However, in AM-inoculated plants, the levels of these oxidative stress markers were significantly lower than those in NM plants under high-iron stress (Figure 6). Specifically, under high-iron stress, AM inoculation reduced shoot MDA content by 39.9%, O_2_^•−^ production rate by 36.4%, and H_2_O_2_ content by 26.7% compared to NM plants; similar trends were observed in roots, with reductions of 31.9%, 31.3%, and 25.1%, respectively. These results provide direct evidence that AM symbiosis effectively alleviates iron-stress-induced oxidative damage by reducing ROS accumulation and lipid peroxidation.

### 2.6. Effects of AM Fungi on Antioxidant Enzyme Activities in Eucalyptus grandis Under Iron Stress

**Figure 7:**
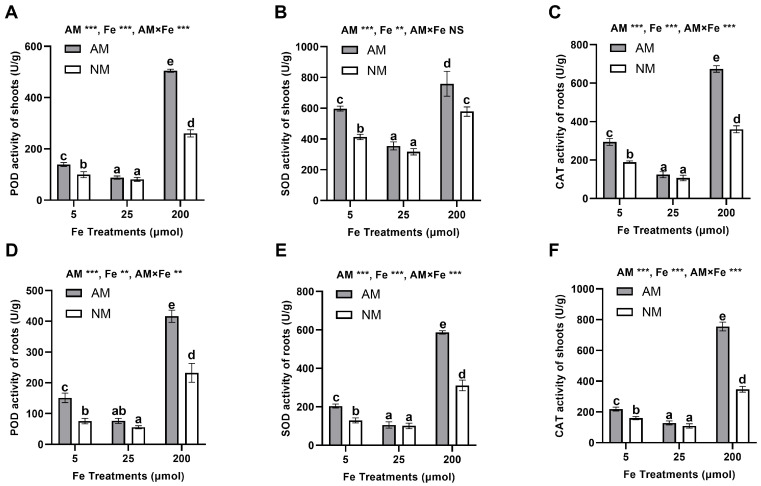
Effects of AM inoculation and iron stress on antioxidant enzyme activities and oxidative stress markers in leaves of *E. grandis*. Peroxidase (POD) activity of shoots (**A**) and roots (**D**); Superoxide dismutase (SOD) activity of shoots (**B**) and roots (**E**); Catalase (CAT) activity of shoots (**C**) and roots (**F**). Different letters indicate significant differences (two-way ANOVA, Tukey’s test, *p* < 0.05). Data are means ± SE (*n* = 3). Significant effect: **: *p*  <  0.01, ***: *p*  <  0.001, NS: no significant effect.

Environmental stresses are often accompanied by excessive production of reactive oxygen species (ROS), and the antioxidant enzyme system serves as a key defense mechanism for plants to scavenge ROS and mitigate oxidative damage. To investigate the effects of AM symbiosis on the antioxidant defense system of E. grandis under iron stress, we measured the activities of peroxidase (POD), superoxide dismutase (SOD), and catalase (CAT) in shoots and roots. At the control iron concentration, there were no significant differences in the activities of these three antioxidant enzymes between AM and NM plants across all tissues. Low iron stress induced an increase in enzyme activity, with AM-inoculated plants showing significantly higher levels than NM-inoculated plants. High-iron stress exerted a more intense oxidative stress effect on the plants; the increase in the activity of the three antioxidant enzymes in the shoots and roots of NM-inoculated plants was greater than that under low iron treatment, while the enzyme activity in AM-inoculated plants rose even further, significantly exceeding that of the corresponding NM-inoculated plants (Figure 7). The response patterns of antioxidant enzyme activities in shoots and roots were consistent. These results indicate that AM fungal colonization significantly enhanced the antioxidant defense capacity of *E. grandis* under both low-iron and high-iron stress conditions, with the enhancing effect being more pronounced under high-iron stress.

### 2.7. Transcriptomic Analysis and qRT-PCR Validation of MYB Transcription Factor Expression

**Figure 8:**
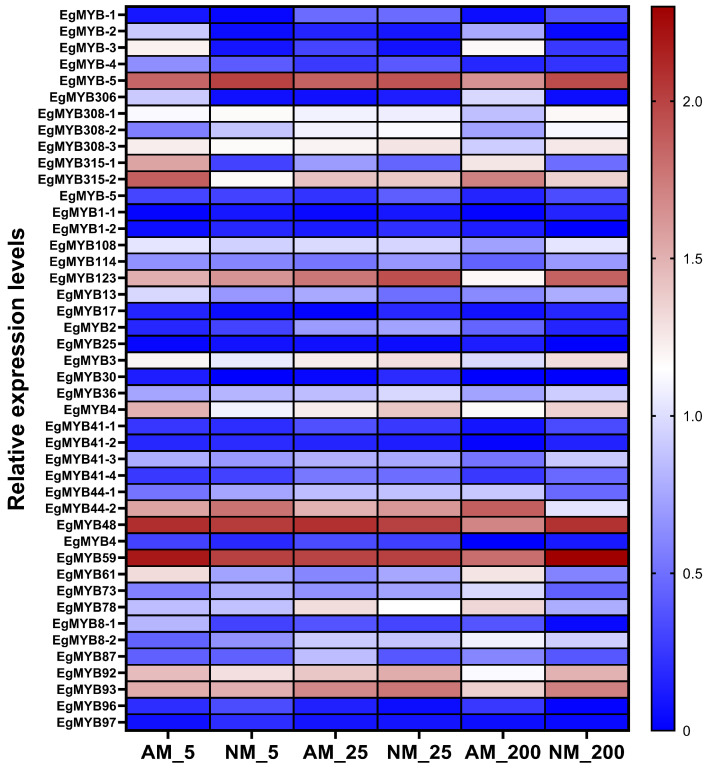
Heatmap analysis of MYB-related transcription factors in *E. grandis* roots under different iron concentrations and AM inoculation conditions. Expression values were derived from RNA-seq data and calculated as log_2_(FPKM + 1). Blue represents low expression, red represents high expression. Samples are arranged as follows: AM_5, NM_5, AM_25, NM_25, AM_200, NM_200.

To further elucidate the molecular mechanisms by which AM fungi regulate the adaptation of E. grandis to iron stress, we analyzed the expression patterns of MYB family transcription factors in the roots of E. grandis under different iron concentrations and AM inoculation treatments, based on transcriptomic sequencing data. The heatmap was plotted using expression values converted to log_2_(FPKM + 1), with red indicating high expression and blue indicating low expression. Cluster analysis revealed that under low iron (5 µM) or high iron (200 µM) conditions, a total of 44 MYB family transcription factors were significantly induced by AM fungi (FDR-corrected, *p* < 0.05) (Figure 8).

To provide broader biological context for the transcriptomic changes, we performed Gene Ontology (GO) enrichment analysis on the differentially expressed genes (DEGs) identified from the transcriptomic data (Supplementary Figure S2). DEGs were significantly enriched in biological processes related to “defense response” (GO:0006952, 450 genes, Padj = 1.77 × 10^−41^), “response to stress” (GO:0006950, 659 genes, Padj = 3.49 × 10^−31^), and “response to oxidative stress” (GO:0006979, 62 genes, Padj = 0.0316), which are consistent with the physiological responses observed under iron stress. Additionally, DEGs were enriched in “photosynthesis, light harvesting” (GO:0009765, 12 genes, Padj = 1.0 × 10^−4^) and “iron ion binding” (GO:0005506, 121 genes, Padj = 0.036), supporting the involvement of photosynthetic protection and iron homeostasis in AM-mediated stress alleviation. Notably, DEGs were also significantly enriched in “DNA-binding transcription factor activity” (GO:0003700, 208 genes, Padj = 3.94 × 10^−5^), indicating that AM symbiosis induces extensive transcriptional reprogramming under iron stress.

Following the selection criteria described above, six MYB genes (*EgMYB-2*, *EgMYB-3*, *EgMYB315-1*, *EgMYB315-2*, *EgMYB61*, and *EgMYB306*) were significantly upregulated by AM fungi under both low-iron and high-iron stress conditions; these genes may participate in the AM-mediated iron stress tolerance regulatory network.

**Figure 9:**
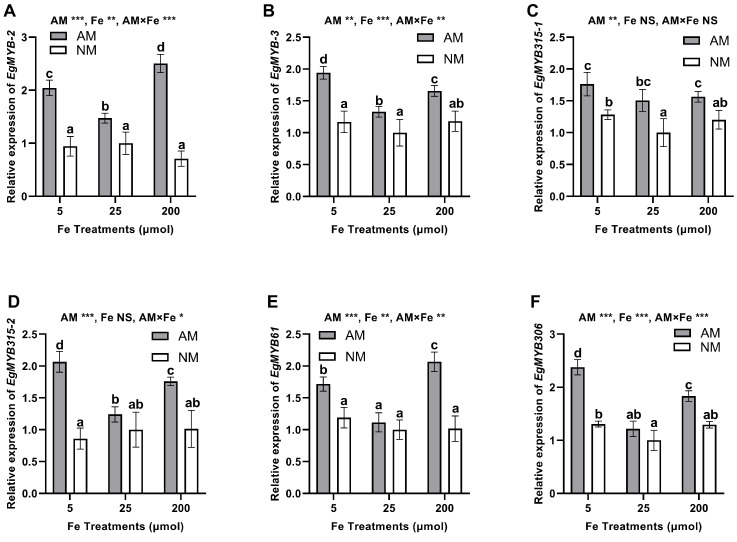
Expression patterns of MYB-related genes in roots of *E. grandis* under different iron supplies and AM inoculation. (**A**) *EgMYB-2*, (**B**) *EgMYB-3*, (**C**) *EgMYB315-1*, (**D**) *EgMYB315-2*, (**E**) *EgMYB61*, (**F**) *EgMYB306* relative expression levels (normalized to *EgUBI3*). NM, non-mycorrhizal; AM, mycorrhizal; Low Fe (5 µM), Control Fe (25 µM), High Fe (200 µM). Different letters indicate significant differences (two-way ANOVA, Tukey’s test, *p* < 0.05). Data are means ± SE (*n* = 3 biological replicates). Significant effect: *: *p*  <  0.05, **: *p*  <  0.01, ***: *p*  <  0.001, NS: no significant effect.

To validate the reliability of the transcriptomic data and further analyze the expression patterns of the aforementioned six MYB genes, we used qRT-PCR to analyze their expression levels under different iron concentrations and AM inoculation treatments (Figure 9). Based on their expression patterns, these genes can be classified into two groups. The first group includes *EgMYB-2*, *EgMYB-3*, and *EgMYB315-1*, whose expression levels in AM-inoculated plants were significantly higher than those in corresponding NM plants at all three iron concentrations (5, 25, and 200 µM) (Figure 9A–C). The second group includes *EgMYB315-2*, *EgMYB61*, and *EgMYB306*. At the control iron concentration (25 µM), there was no significant difference between AM and NM plants; however, under low- and high-iron stress conditions, the expression of these three genes was significantly upregulated in AM plants, showing a substantial increase compared to NM plants (Figure 9D–F). These results indicate that the regulation of MYB transcription factors by AM fungi is gene-specific and iron-concentration-dependent; these MYB genes may collaboratively participate in the adaptive regulatory network of *E. grandis* under iron stress.

### 2.8. Correlation Analysis of MYB Gene Expression with Physiological Parameters

**Figure 10:**
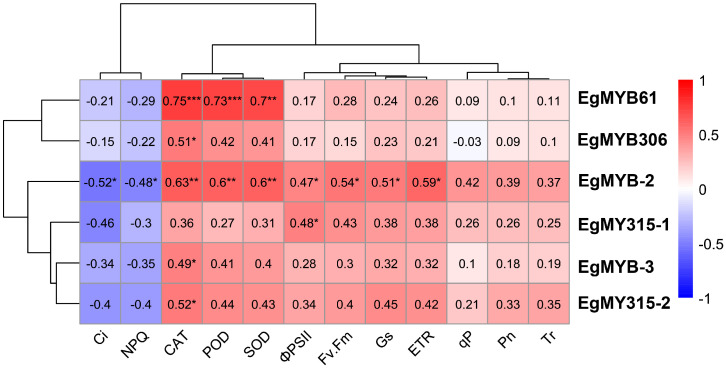
Correlation heatmap among photosynthetic parameters, chlorophyll fluorescence parameters, antioxidant enzyme activities, and MYB gene expression in *E. grandis* under combined iron and AM treatments. The heatmap displays Pearson correlation coefficients (r) with significance labels (* *p* < 0.05, ** *p* < 0.01, *** *p* < 0.001). Red indicates positive correlation; blue indicates negative correlation. Hierarchical clustering was applied to both rows (MYB genes) and columns (physiological parameters) using Euclidean distance and complete linkage method. Abbreviations: Pn, net photosynthetic rate; Gs, stomatal conductance; Ci, intercellular CO_2_ concentration; Tr, transpiration rate; Fv/Fm, maximum quantum yield of PSII; ΦPSII, actual photochemical efficiency of PSII; ETR, electron transport rate; qP, photochemical quenching; NPQ, non-photochemical quenching; SOD, superoxide dismutase; POD, peroxidase; CAT, catalase; *EgMYB-2*, *EgMYB-3*, *EgMYB315-1*, *EgMYB315-2*, *EgMYB61*, *EgMYB306*. *n* = 18 biological samples (3 biological replicates × 2 inoculation treatments × 3 iron concentrations).

To investigate the potential associations between MYB transcription factors and physiological responses under AM-mediated iron stress tolerance, we performed Pearson correlation analysis between the expression levels of the six AM-induced MYB genes and the measured physiological parameters, including antioxidant enzyme activities (POD, SOD, CAT), photosynthetic parameters (Pn, Gs, Ci, Tr), and chlorophyll fluorescence parameters (Fv/Fm, ΦPSII, ETR, qP, NPQ) (Figure 10). The correlation analysis revealed that *EgMYB61* exhibited the strongest correlations with antioxidant enzyme activities, showing significant positive correlations with CAT (r = 0.75, *p* < 0.001), POD (r = 0.73, *p* < 0.001), and SOD (r = 0.70, *p* < 0.01). *EgMYB-2* showed moderate positive correlations with antioxidant enzymes (CAT: r = 0.63; POD: r = 0.60; SOD: r = 0.60) and stronger correlations with photosynthetic parameters including ETR (r = 0.59, *p* < 0.05), Fv/Fm (r = 0.54, *p* < 0.05), Gs (r = 0.51, *p* < 0.05), and ΦPSII (r = 0.47, *p* < 0.05). Its expression was negatively correlated with Ci (r = −0.52, *p* < 0.05) and NPQ (r = −0.48, *p* < 0.05). *EgMYB315-2* was specifically correlated with CAT activity (r = 0.52, *p* < 0.05) and also showed moderate positive correlations with POD, SOD, Gs, and CAT. *EgMYB315-1* showed the strongest correlation with ΦPSII (r = 0.48, *p* < 0.05) and moderate correlations with Fv/Fm (r = 0.43), Gs (r = 0.38), ETR (r = 0.38), and CAT (r = 0.36). *EgMYB-3* exhibited moderate positive correlations with CAT (r = 0.49), SOD (r = 0.40), and POD (r = 0.41), while *EgMYB306* showed the weakest correlations with most parameters (r = −0.03 to 0.51), with only CAT reaching moderate correlation (r = 0.51).

## 3. Discussion

The role of arbuscular mycorrhizal fungi in improving plant mineral nutrition and alleviating abiotic stress has been confirmed in numerous plant species [37,38]. However, research on the mechanisms by which they regulate iron nutrition in forest tree species remains relatively limited. This study systematically investigated the effects of the AM fungus *R. irregularis* on the growth, photosynthetic performance, antioxidant defense systems, and MYB transcription factor expression of *E. grandis* under different iron supply levels, providing new evidence for understanding how AM symbiosis regulates forest tree adaptation to iron stress.

### 3.1. AM Fungi Enhance Photosynthetic Performance and Regulate Iron Accumulation

Iron is an indispensable factor in plant photosynthesis, respiration, and chlorophyll synthesis; both its deficiency and excess can severely inhibit plant growth [3,39]. Consistent with previous reports [40], our study observed that low-iron (5 µM) and high-iron (200 µM) stress significantly inhibited the growth and photosynthetic performance of NM *E. grandis*, while AM inoculation significantly alleviated these inhibitory effects, as also observed in sunflower [18]. Similar growth promotion has also been reported in woody species such as Populus and Citrus under heavy metal stress [41,42]. A key finding of this study is the association between AM symbiosis and plant Fe homeostasis: under low-iron conditions, AM inoculation significantly increased shoot and root Fe content, while under high-iron conditions, it significantly reduced Fe accumulation (Figure 3), suggesting that AM symbiosis may act as a modulator of Fe availability under both deficiency and toxicity.

The maintenance of photosynthetic efficiency is a key physiological basis for AM-mediated plant growth promotion. Iron deficiency leads to alterations in membrane structure and reduced PSII efficiency, while also disrupting iron-dependent chlorophyll biosynthesis pathways [43]. Under both stress conditions, Pn, Gs, and Fv/Fm were significantly higher in AM plants than in NM plants (Figure 4 and Figure 5), suggesting that AM symbiosis was associated with enhanced PSII photochemical activity, consistent with findings in sunflower [18]. The lower Ci combined with higher Gs in AM plants suggests that photosynthetic limitation in NM plants primarily stems from stomatal factors rather than mesophyll damage, consistent with findings in drought-stressed plants [44] and *Populus cathayana* under Cd stress [45]. The increased chlorophyll content in AM plants further supports this conclusion [46]. Similar PSII protection by AM fungi has been reported in *Lactuca sativa* under drought stress [47].

### 3.2. AM Fungi Improve Antioxidant Defense and Reduce Oxidative Damage in Plants Under Iron Stress

Abiotic stresses can induce excessive production of reactive oxygen species (ROS), leading to oxidative stress. Iron serves as a cofactor or structural component of antioxidant enzymes such as SOD, CAT, and APX [48,49,50]; therefore, iron imbalance has a particularly direct impact on the antioxidant system. In sunflowers, AM fungi inoculation significantly enhanced the activities of CAT, SOD, and glutathione reductase (GR) in the root system under iron-deficient conditions, indicating a close association between AM fungi and the induction of antioxidant enzymes [18]. In this study, AM inoculation significantly enhanced POD, SOD, and CAT activities under both stress conditions (Figure 7), consistent with observations in other species under metal stress [51,52,53], with greater increases observed under high-iron stress than under low-iron stress. More importantly, the reduced accumulation of MDA, O_2_^−^, and H_2_O_2_ in AM plants (Figure 6) provides direct evidence that AM symbiosis effectively mitigated oxidative damage. This dual effect—stimulation of antioxidant enzymes and reduction in ROS—may represents a key mechanism by which AM fungi alleviate iron stress in woody species. Future studies could further elucidate the transcriptional regulation of antioxidant defense by analyzing the expression of genes encoding SOD, POD, and CAT in response to AM inoculation under iron stress.

### 3.3. MYB Transcription Factors Associated with AM Symbiosis in the Adaptation of Eucalyptus grandis to Iron Stress

In response to iron stress, plants must activate a complex transcriptional regulatory network, in which members of the MYB transcription factor family play a key role in responses to abiotic stress. MYB transcription factors are extensively involved in processes such as secondary metabolism, cell morphogenesis, hormone signal transduction, and stress responses [27,54,55]. In *Arabidopsis*, AtMYB10, AtMYB72, and AtMYB59 have been shown to participate in the regulation of the iron-deficiency response; they activate or suppress target gene expression by directly binding to cis-acting elements in the promoter regions of downstream genes [28,29,56]. It is reported that MYB8 regulates iron deficiency stress response and MYB30 promotes iron homeostasis by maintaining the stability of the FIT transcription factor in *Arabidopsis* [57,58]. In woody species, MYB functions in Fe stress have been reported in *Malus halliana* [31] and *Malus xiaojinensis* [32]. Our findings extend these observations to *E. grandis* and suggest that AM symbiosis is associated with the upregulation of specific MYB genes under both low and high iron stress conditions.

Based on transcriptomic data, we identified 44 AM-induced MYB transcription factors, among which *EgMYB-2*, *EgMYB-3*, *EgMYB315-1*, *EgMYB315-2*, *EgMYB61*, and *EgMYB306* were significantly upregulated by AM under both low- and high-iron conditions. Based on their expression patterns, these MYB genes can be classified into two categories: the first category (*EgMYB-2*, *EgMYB-3*, and *EgMYB315-1*) is induced by AM under all three iron concentrations, suggesting their involvement in the regulation of defenses underlying AM symbiosis; the second group (*EgMYB315-2*, *EgMYB61*, and *EgMYB306*) is induced by AM only under iron-stress conditions, indicating that they are specifically involved in stress responses, suggesting that AM symbiosis coordinates the plant integrated response to iron stress through multi-level transcriptional regulatory networks. Homology analysis revealed that these genes are associated with ABA-dependent stress responses (*EgMYB-2/3*), metal ion homeostasis (*EgMYB315-1/2*), lignin biosynthesis (*EgMYB61*), and oxidative stress responses (*EgMYB306*). The identification of both constitutively AM-induced MYB genes and stress-specific MYB genes suggests a hierarchical transcriptional regulatory network.

Correlation analysis further revealed that the six MYB genes exhibited distinct functional differentiation (Figure 10). Among them, *EgMYB61* showed the strongest correlations with antioxidant enzymes (CAT, POD and SOD), suggesting its potential role as a key regulator of antioxidant defense under iron stress. In contrast, *EgMYB-2* exhibited broader correlations with both antioxidant enzymes and photosynthetic parameters, indicating a more general regulatory function, while *EgMYB315-2* and *EgMYB315-1* showed more specialized correlations with CAT and ΦPSII, respectively. The weak correlations observed for *EgMYB306* suggest that this gene may be involved in other aspects of stress responses not assessed in this study. These functional differentiation patterns are consistent with previous studies in *Arabidopsis* and woody species, where different MYB transcription factors have been shown to play distinct roles in stress responses. In *Arabidopsis*, AtMYB10, AtMYB72, and AtMYB59 specifically regulate iron-deficiency responses [28,29,54]. In *Malus halliana* and *Malus xiaojinensis*, MYB genes also exhibited functional specialization in Fe stress responses [31,32]. The constitutive versus stress-specific expression patterns of our six MYB genes further suggest a multi-layered regulatory strategy, where constitutively AM-induced MYB genes (e.g., *EgMYB-2*) may maintain basal stress preparedness, while stress-inducible genes (e.g., *EgMYB61*) provide additional regulation under severe stress conditions. These correlations provide robust candidate targets for future functional studies, and the distinct association patterns observed among the six MYB genes highlight the complexity of AM-mediated transcriptional regulation under iron stress. While these findings do not establish direct regulatory relationships, they offer a solid foundation for hypothesis-driven validation through transgenic approaches, such as yeast one-hybrid assays or stable transformation in *Eucalyptus*. Moreover, WGCNA or broader network analysis with larger sample sizes and time-course sampling in future studies could further elucidate the gene modules associated with iron stress.

On the basis of these findings, we propose a putative regulatory pathway: mycorrhizal colonization is associated with differential Fe uptake, which correlates with protection of the photosynthetic apparatus and PSII function; simultaneously, AM symbiosis is associated with enhanced antioxidant defense; these physiological changes are accompanied by transcriptional reprogramming of MYB genes that may coordinate adaptive responses to iron stress. Future studies employing transgenic validation of candidate MYB genes (particularly *EgMYB61* and E*gMYB-2*), Fe speciation analysis, and PSI activity and OJIP analysis will be necessary to further elucidate these mechanisms and their practical applications.

## 4. Materials and Methods

### 4.1. Plant Materials and Fungal Strains

*Eucalyptus grandis* seeds were provided by the Institute of Tropical Forestry, Chinese Academy of Forestry Sciences. The arbuscular mycorrhizal fungus used in the experiment was *Rhizophagus irregularis* (strain DAOM 197198), purchased from Agronutrition in France.

### 4.2. Growing Medium and Plant Cultivation

A mixture of quartz sand and vermiculite in a 3:1 (*v*/*v*) ratio was used as the growing medium. Prior to use, the medium was autoclaved at 121 °C for 2 h. Seeds of E. grandis were surface-disinfected with a 3% (*v*/*v*) sodium hypochlorite solution for approximately 15 min, rinsed five times with sterile water, and placed in 1/2 MS medium for germination at 25 °C in the dark. After germination, the seedlings were transferred to a growth environment with 16 h of light (26 °C and 8 h of darkness (22 °C) and cultured for 2 weeks. Seedlings of uniform growth were selected and transplanted into plastic pots (8 cm × 8 cm × 8 cm), each filled with 500 g of sterilized substrate.

Approximately 500 spores were inoculated into each pot (AM treatment); an equal volume of deionized water was added to the non-mycorrhizal (NM) treatment. The inoculum consisted of spores mixed with sterilized sand carrier, with a spore purity of >95% and viability of >90% as determined by MTT (3-(4,5-dimethylthiazol-2-yl)-2,5-diphenyl-2H-razolium bromide) staining assay [59]. Six pots were used for each treatment. After transplanting, each pot was watered with 50 mL of modified Long-Ashton (mLA) nutrient solution (with EDTA-Fe^3+^ as the iron source) [60]. The mLA nutrient solution contained the following macronutrients: NaNO_3_ (1 mM), CaCl_2_·2H_2_O (2 mM), MgSO_4_·7H_2_O (0.75 mM), NaH_2_PO_4_ (0.3 mM), K_2_SO_4_ (1 mM); micronutrients: H_3_BO_3_ (0.025 mM), Na_2_MoO_4_·2H_2_O (0.0001 mM), CuSO_4_·5H_2_O (0.00025 mM), ZnSO_4_·7H_2_O (0.0005 mM), MnSO_4_·H_2_O (0.005 mM) and FeNa·EDTA (0.025 mM).

### 4.3. Iron Concentration Treatments

Iron concentration treatments began in the fourth week after transplanting. Based on the basal nutrient solution, three Fe concentrations were established: Low Fe: 5 µM; Control Fe: 25 µM; High Fe: 200 µM. For each treatment, 50 mL of the corresponding nutrient solution was applied to each pot once every 3 days for 4 consecutive weeks. The entire experiment was conducted in a climate-controlled chamber under a 16/8 h (day/night) photoperiod, at temperatures of 26/22 °C and relative humidity of 65%.

### 4.4. Harvesting and Sample Collection

Plant samples were collected 4 weeks after the iron treatments. After measuring plant height and root length, the aboveground parts were separated from the root system. A portion of the fresh roots was immediately used for mycorrhizal colonization rate determination; the remaining samples were rapidly frozen in liquid nitrogen and stored at −80 °C for subsequent gene expression and enzyme activity analyses. There were three independent biological replicates for each treatment. In each replicate, six seedlings were pooled and evenly divided into three subsamples for parallel measurements. These replicates were used for physiological parameter measurements, enzyme activity assays, transcriptome sequencing, and qRT-PCR validation.

### 4.5. Determination of Mycorrhizal Colonization

The mycorrhizal colonization was determined using the trypan blue staining method. Fresh fine root segments were placed in a 5% KOH (*w*/*v*) solution and decolorized in a 90 °C water bath for 30 min. After rinsing once with distilled water, the roots were acidified in a 1% HCl solution at room temperature for 10 min. Subsequently, they were stained with a 0.05% trypan blue solution in a 90 °C water bath for 30 min [61]. Finally, decolorize the root segments in a lactic acid–glycerol (1:1, *v*:*v*) decolorizing solution in a 90 °C water bath for 30 min. Prepare slides from the decolorized root segments and use the MYCOCALC program to quantitatively analyze the mycorrhizal infection rate [62], while observing and photographing AM fungal structures under an optical microscope (Olympus BX51, Olympus Corporation, Tokyo, Japan).

### 4.6. Determination of Iron Content

To determine iron concentrations in plant tissues, dried shoot and root samples (about 0.5 g) were ground to a fine powder and digested with a mixture of HNO_3_ and HClO_4_ (4:1, *v*/*v*) at 180 °C until the digest became clear. The digested solution was diluted to 25 mL with deionized water, and iron content was measured using an inductively coupled plasma optical emission spectrometer (ICP-OES, Thermo Scientific iCAP 7000, Thermo Fisher Scientific, Waltham, MA, USA) [63]. Iron concentrations were expressed as mg·g^−1^ dry weight. Three biological replicates were analyzed per treatment.

### 4.7. Measurement of Photosynthetic Parameters

One day before harvest, the third fully expanded leaf from the apex of 8-week-old plants (4 weeks after iron treatment initiation) was selected. Between 9:30 and 11:30 a.m., the net photosynthetic rate (Pn), stomatal conductance (Gs), intercellular CO_2_ concentration (Ci), and transpiration rate (Tr) were measured using a LI-6400XT portable photosynthetic analyzer (Li-Cor, Lincoln, NE, USA). During the measurements, light intensity was set to 1000 µmol·m^−2^·s^−1^, CO_2_ concentration to 400 µmol·mol^−1^, and temperature to 25 °C.

### 4.8. Measurement of Chlorophyll Fluorescence Parameters

Prior to harvest, chlorophyll fluorescence parameters were measured using a Mini-Imaging-Pam fluorometer (Walz, Nuremberg, Germany) in accordance with the manufacturer’s instructions. Leaves were dark-adapted for 30 min using leaf clips before measurements, and this procedure was applied identically to all treatments. The measured parameters included: maximum photochemical quantum yield of PSII (Fv/Fm), effective photochemical efficiency of PSII (ΦPSII), photochemical quenching coefficient (qP), and non-photochemical quenching coefficient (NPQ).

### 4.9. Determination of Antioxidant Enzyme Activity

Approximately 0.3 g of shoots and root samples were weighed and placed in a pre-chilled mortar, to which 3 mL of 50 mM phosphate buffer (pH 7.8, containing 1% PVP) was added and ground to a homogenate on an ice bath. The homogenate was centrifuged at 10,000× *g* for 20 min at 4 °C; the supernatant was the crude enzyme solution. Superoxide dismutase (SOD) activity was determined using the nitroblue tetrazolium (NBT) photoreduction method, with 50% inhibition of NBT photoreduction defined as one unit of enzyme activity. Peroxidase (POD) activity was determined using the guaiacol method, measuring changes in absorbance at 470 nm. Catalase (CAT) activity was determined using the UV absorption method at 240 nm [64,65,66]. Three biological replicates were set up for each treatment.

### 4.10. Determination of Lipid Peroxidation and Reactive Oxygen Species

Lipid peroxidation was estimated by measuring malondialdehyde (MDA) content using the thiobarbituric acid (TBA) method [67]. Fresh samples were homogenized in trichloroacetic acid (TCA), and the supernatant was reacted with TBA. Absorbance was measured at 532 nm, 600 nm, and 450 nm, and MDA content was calculated using the formula of Heath and Packer.

Superoxide anion (O_2_^−^) production rate was determined using the hydroxylamine oxidation method [68]. Fresh samples were homogenized in phosphate buffer and incubated with hydroxylamine hydrochloride, followed by the addition of p-aminobenzenesulfonic acid and α-naphthylamine. Absorbance was measured at 530 nm, and O_2_^−^ production rate was calculated using a NaNO_2_ standard curve.

Hydrogen peroxide (H_2_O_2_) content was determined using the titanium sulfate method. Fresh samples were homogenized in cold acetone, and the extract was reacted with titanium sulfate. The absorbance was measured at 415 nm, and H_2_O_2_ content was calculated using a standard curve [69]. Three biological replicates were analyzed per treatment.

### 4.11. Total RNA Extraction and qRT-PCR Validation

Total RNA was extracted from leaves and roots using the Plant RNA Kit (Omega Bio-Tek, Norcross, GA, USA). RNA integrity and purity were assessed via 1% agarose gel electrophoresis and a NanoDrop 2000 spectrophotometer (Thermo Scientific, Wilmington, DE, USA). The first strand of cDNA was synthesized via reverse transcription using the PrimeScript™ RT Reagent Kit with gDNA Eraser (Takara, Kusatsu, Shiga, Japan). Specific primers for the target MYB genes (selected based on transcriptomic data) were designed using Primer Premier 5.0. The primer sequences are listed in Supplementary Table S1. *EgUBI3* (ubiquitin ligase gene) was used as the internal control. qRT-PCR reactions were performed on a Bio-Rad CFX96 real-time quantitative PCR system using ChamQ Universal SYBR qPCR Master Mix (Vazyme, Nanjing, China). The reaction volume was 20 µL (containing 10 µL SYBR Green Master Mix, 0.2 µM each of forward and reverse primers, 2 µL cDNA, and ddH_2_O to a total volume of 20 µL). Amplification program: 3 min pre-denaturation at 95 °C; denaturation at 95 °C for 15 s, annealing at 60 °C for 30 s, and extension at 72 °C for 30 s, for 40 cycles; this was followed by melting curve analysis to verify amplification specificity. Each sample included 3 biological replicates and 3 technical replicates, and relative expression levels were calculated using the 2^−ΔΔCt^ method.

### 4.12. Transcriptome Sequencing and Data Analysis

Total RNA samples from the roots of *E. grandis* under different iron concentrations and AM inoculation treatments were sent to a sequencing company for transcriptome sequencing. Paired-end sequencing (150 bp) was performed using the Illumina NovaSeq platform. The sequencing generated approximately 12 Gb of clean data per sample, with Q30 scores >92%. Raw sequencing data were filtered using Trimmomatic (v0.39) to remove low-quality reads and adapter sequences, then aligned to the *E. grandis* reference genome [34] using HISAT2 (v2.1.0), with a mapping efficiency of >85%. Gene expression levels were calculated using feature Counts (v2.0.1) and normalized to FPKM (Fragments Per Kilobase of transcript per Million mapped reads). Differentially expressed gene analysis was performed using DESeq2 (v1.34.0), with a significance threshold of |log_2_FC| ≥ 1, average FPKM ≥ 1, and an FDR-corrected *p* < 0.05 (Benjamini–Hochberg test). For genome-wide identification of MYB transcription factor family members, protein sequences of the *E. grandis* genome were searched against the Pfam MYB domain (PF00249) using HMMER (v3.3) with an E-value cutoff of 1e^−5^; candidate sequences were further verified by the presence of the conserved R2R3-MYB domain using SMART and NCBI-CDD [70,71]. Heatmaps were generated using CLC Genomics Workbench (v20) software based on expression values transformed as log_2_(FPKM + 1).

For the selection of MYB genes for qRT-PCR validation from the 44 differentially expressed MYB transcription factors, the following criteria were applied: (1) consistent differential expression between AM and NM treatments under both low- and high-iron stress conditions; (2) |log_2_FC| ≥ 2 (fold change ≥ 4) between AM and NM samples; and (3) average FPKM ≥ 10 to ensure reliable expression detection.

### 4.13. Statistical Analysis

Two-way analysis of variance (ANOVA) was performed using SPSS 22.0 software, with factors including AM inoculation (AM/NM) and iron concentration (5, 25, 200 µM). Tukey’s test was used for multiple comparisons (*p* < 0.05). Redundancy analysis (RDA) was performed using R (v4.1.0), and graphs were plotted using the ggplot2 package (v3.3.5). Data in the figures are presented as mean ± standard error (SE).

## 5. Conclusions

This study demonstrates that AM symbiosis enhances iron stress tolerance in *E. grandis* through a multi-level synergistic strategy integrating regulation of Fe uptake, photosynthetic protection, antioxidant defense activation, and transcriptional reprogramming of MYB transcription factors. A key finding is the dual functionality of AM symbiosis—promoting Fe acquisition under deficiency while restricting excessive accumulation under toxicity. The identification of six AM-induced MYB genes (*EgMYB-2*, *EgMYB-3*, *EgMYB315-1*, *EgMYB315-2*, *EgMYB61*, and *EgMYB306*) provides candidate targets for future functional studies, and the correlation between their expression and physiological parameters suggests their potential role as regulatory hubs coordinating stress responses, with *EgMYB61* showing the strongest association with antioxidant defense and *EgMYB-2* potentially coordinating both antioxidant and photosynthetic pathways. Future research should focus on functional validation of these MYB genes through transgenic approaches, investigation of Fe speciation, and field-scale evaluation of AM inoculation efficacy. From a practical perspective, this study provides a theoretical basis for the application of mycorrhizal biotechnology in *E. grandis* cultivation on iron-stressed sites, offering a sustainable strategy to improve seedling establishment and growth in challenging soil environments.

## Data Availability

The data presented in this study are available on request from the corresponding author. The data are not publicly available due to ongoing research and further data analysis.

## Supplementary Materials

The following supporting information can be downloaded at: https://www.mdpi.com/article/10.3390/plants15142213/s1.

## References

[1] Fan Z., Wu Y., Zhao L., Fu L., Deng L., Deng J., Ding D., Xiao S., Deng X., Peng S.A. (2022). MYB308-mediated transcriptional activation of plasma membrane H^+^-ATPase 6 promotes iron uptake in citrus. Hortic. Res..

[2] Johnson L. (2008). Iron and siderophores in fungal–host interactions. Mycol. Res..

[3] Zhang X., Zhang D., Sun W., Wang T. (2019). The Adaptive Mechanism of Plants to Iron Deficiency via Iron Uptake, Transport, and Homeostasis. Int. J. Mol. Sci..

[4] Varotto C., Maiwald D., Pesaresi P., Jahns P., Salamini F., Leister D. (2002). The metal ion transporter IRT1 is necessary for iron homeostasis and efficient photosynthesis in Arabidopsis thaliana. Plant J..

[5] Lucena J.J., Hernandez-Apaolaza L. (2017). Iron nutrition in plants: An overview. Plant Soil.

[6] Walker E.L., Connolly E.L. (2008). Time to pump iron: Iron-deficiency-signaling mechanisms of higher plants. Curr. Opin. Plant Biol..

[7] Colombo C., Palumbo G., He J., Pinton R., Cesco S. (2014). Review on iron availability in soil: Interaction of Fe minerals, plants, and microbes. J. Soils Sediments.

[8] López-Lorca V.M., López-Castillo O., Molina-Luzón M.J., Ferrol N. (2026). Arbuscular Mycorrhiza Modulates Iron Distribution and Vacuolar Iron Transporter Expression in Tomato, Whereas Iron Limitation Reduces Mycorrhization. Plant Cell Environ..

[9] Smith S.E., Read D.J. (2008). Mycorrhizal Symbiosis.

[10] Lanfranco L., Fiorilli V., Gutjahr C. (2018). Partner communication and role of nutrients in the arbuscular mycorrhizal symbiosis. New Phytol..

[11] Wang W., Shi J., Xie Q., Jiang Y., Yu N., Wang E. (2017). Nutrient Exchange and Regulation in Arbuscular Mycorrhizal Symbiosis. Mol. Plant.

[12] Wang S., Xie X., Che X., Lai W., Ren Y., Fan X., Hu W., Tang M., Chen H. (2023). Host- and virus-induced gene silencing of HOG1-MAPK cascade genes in *Rhizophagus irregularis* inhibit arbuscule development and reduce resistance of plants to drought stress. Plant Biotechnol. J..

[13] Wang S., Han L., Ren Y., Hu W., Xie X., Chen H., Tang M. (2024). The receptor kinase RiSho1 in *Rhizophagus irregularis* regulates arbuscule development and drought tolerance during arbuscular mycorrhizal symbiosis. New Phytol..

[14] Jenab K., Alteio L., Guseva K., Gorka S., Darcy S., Fuchslueger L., Canarini A., Martin V., Wiesenbauer J., Spiegel F. (2026). Arbuscular mycorrhizal fungal families and exploration-based guilds exhibit distinct responses to long-term N, P and K deficiencies and imbalances. New Phytol..

[15] Li J., He X., Li H., Zheng W., Liu J., Wang M. (2015). Arbuscular mycorrhizal fungi increase growth and phenolics synthesis in Poncirus trifoliata under iron deficiency. Sci. Hortic..

[16] Rajapitamahuni S., Kang B.R., Lee T.K. (2023). Exploring the Roles of Arbuscular Mycorrhizal Fungi in Plant–Iron Homeostasis. Agriculture.

[17] Szentpéteri V., Mayer B., Posta K. (2024). The role of arbuscular mycorrhizal fungi in the alleviation of iron deficiency stress in plants. Plants.

[18] Kabir A.H., Debnath T., Das U., Prity S.A., Haque A., Rahman M.M., Parvez M.S. (2020). Arbuscular mycorrhizal fungi alleviate Fe-deficiency symptoms in sunflower by increasing iron uptake and its availability along with antioxidant defense. Plant Physiol. Biochem..

[19] Ferrol N., Tamayo E., Vargas P. (2016). The heavy metal paradox in arbuscular mycorrhizas: From mechanisms to biotechnological applications. J. Exp. Bot..

[20] Mittler R., Zandalinas S.I., Fichman Y., Van Breusegem F. (2022). Reactive oxygen species signalling in plant stress responses. Nat. Rev. Mol. Cell Biol..

[21] Dumanović J., Nepovimova E., Natić M., Kuča K., Jaćević V. (2021). The Significance of Reactive Oxygen Species and Antioxidant Defense System in Plants: A Concise Overview. Front. Plant Sci..

[22] Rao M.J., Duan M., Zhou C., Jiao J., Cheng P., Yang L., Wei W., Shen Q., Ji P., Yang Y. (2025). Antioxidant Defense System in Plants: Reactive Oxygen Species Production, Signaling, and Scavenging During Abiotic Stress-Induced Oxidative Damage. Horticulturae.

[23] Liu J., Osbourn A., Ma P. (2015). MYB Transcription Factors as Regulators of Phenylpropanoid Metabolism in Plants. Mol. Plant.

[24] Wang X., Zhao S., Zhou R., Liu Y., Guo L., Hu H. (2023). Identification of Vitis vinifera MYB transcription factors and their response against grapevine berry inner necrosis virus. BMC Plant Biol..

[25] Sabir I.A., Manzoor M.A., Shah I.H., Liu X., Zahid M.S., Jiu S., Wang J., Abdullah M., Zhang C. (2022). MYB transcription factor family in sweet cherry (*Prunus avium* L.): Genome-wide investigation, evolution, structure, characterization and expression patterns. BMC Plant Biol..

[26] Pucker B., Pandey A., Weisshaar B., Stracke R. (2020). The R2R3-MYB gene family in banana *(Musa acuminata*): Genome-wide identification, classification and expression patterns. PLoS ONE.

[27] Dubos C., Stracke R., Grotewold E., Weisshaar B., Martin C., Lepiniec L. (2010). MYB transcription factors in *Arabidopsis*. Trends Plant Sci..

[28] Palmer C.M., Hindt M.N., Schmidt H., Clemens S., Guerinot M.L. (2013). MYB10 and MYB72 Are Required for Growth under Iron-Limiting Conditions. PLoS Genet..

[29] Kobayashi T. (2019). Understanding the Complexity of Iron Sensing and Signaling Cascades in Plants. Plant Cell Physiol..

[30] Gong Q. (2026). Metabolic analysis of MYB30 that regulates iron deficiency stress in *Arabidopsis*. Front. Plant Sci..

[31] Zhang Z.X., Zhang R., Wang S.C., Zhang D., Zhao T., Liu B., Wang Y.X., Wu Y.X. (2022). Identification of *Malus halliana* R2R3-MYB gene family under iron deficiency stress and functional characteristics of MhR2R3-MYB4 in *Arabidopsis thaliana*. Plant Biol..

[32] Shen J., Xu X., Li T., Cao D., Han Z. (2008). An MYB transcription factor from *Malus xiaojinensis* has a potential role in iron nutrition. J. Integr. Plant Biol..

[33] Yang X., Guo T., Li J., Chen Z., Guo B., An X. (2021). Genome-wide analysis of the MYB-related transcription factor family and associated responses to abiotic stressors in Populus. Int. J. Biol. Macromol..

[34] Myburg A.A., Grattapaglia D., Tuskan G.A., Hellsten U., Hayes R.D., Grimwood J., Schmutz J. (2014). The genome of *Eucalyptus grandis*. Nature.

[35] He X., Lu Z., Yang J., Cheng F. (2025). Size composition, stability, and distribution of metal nutrient elements of soil aggregates of Eucalyptus plantations with different thinning intensities. Forests.

[36] Assimakopoulou A., Holevas C.D., Fasseas K. (2011). Relative susceptibility of some Prunus rootstocks in hydroponics to iron deficiency. J. Plant Nutr..

[37] Wu Y., Chen C., Wang G. (2024). Inoculation with arbuscular mycorrhizal fungi improves plant biomass and nitrogen and phosphorus nutrients: A meta-analysis. BMC Plant Biol..

[38] Wang M., Xia R., Hu L., Dong T., Wu Q. (2007). Arbuscular mycorrhizal fungi alleviate iron deficient chlorosis in *Poncirus trifoliata* L. Raf under calcium bicarbonate stress. J. Hortic. Sci. Biotech..

[39] Liang G. (2022). Iron uptake, signaling, and sensing in plants. Plant Commun..

[40] Kabir A.H., Paltridge N.G., Roessner U., Stangoulis J.C.R. (2013). Mechanisms associated with Fe-deficiency tolerance and signaling in shoots of *Pisum sativum*. Physiol. Plan..

[41] Xu N., Wei X., Wang Y., Dong J., Yang X. (2025). Mechanism of Arbuscular Mycorrhizal Fungi in Enhancing Lead Stress Resistance in Poplar Trees. Forests.

[42] Meng L.-L., Li C.-Z., Zou B.-W., Zou Y.-N., Srivastava A.K., Wu Q.-S. (2026). Efficacy of Arbuscular Mycorrhizal Fungi in Alleviating Manganese Stress in Trifoliate Orange. Agriculture.

[43] Saito A., Shinjo S., Ito D., Doi Y., Sato A., Wakabayashi Y., Honda J., Arai Y., Maeda T., Ohyama T. (2021). Enhancement of Photosynthetic Iron-Use Efficiency Is an Important Trait of *Hordeum vulgare* for Adaptation of Photosystems to Iron Deficiency. Plants.

[44] Augé R.M., Toler H.D., Saxton A.M. (2015). Arbuscular mycorrhizal symbiosis alters stomatal conductance of host plants more under drought than under amply watered conditions: A meta-analysis. Mycorrhiza.

[45] Chen L., Lai J., Tan L.-J. (2017). Effects of inoculation with arbuscular mycorrhizal fungi on photosynthetic physiology in females and males of *Populus deltoides* exposed to cadmium pollution. Chin. J. Plant Ecol..

[46] de Andrade S.A., Domingues A.P., Mazzafera P. (2015). Photosynthesis is induced in rice plants that associate with arbuscular mycorrhizal fungi and are grown under arsenate and arsenite stress. Chemosphere.

[47] Azcón R., Gómez M., Tobar R. (1995). Influence of different *Glomus* species on the time-course of physiological plant responses of lettuce to progressive drought stress periods. Plant Sci..

[48] Halliwell B. (2006). Reactive Species and Antioxidants. Redox Biology Is a Fundamental Theme of Aerobic Life. Plant Physiol..

[49] Mittler R. (2002). Oxidative stress, antioxidants and stress tolerance. Trends Plant Sci..

[50] Kabir A.H., Rahman M.M., Haider S.A., Paul N.K. (2015). Mechanisms associated with differential tolerance to Fe deficiency in okra (*Abelmoschus esculentus* Moench). Environ. Exp. Bot..

[51] Kumar A., Das S., Pradhan A.K., Pradhan A.K. (2023). Arbuscular mycorrhiza augments aluminum tolerance in white clover (*Trifolium repens* L.) by strengthening the ascorbate–glutathione cycle and phosphorus acquisition. Physiol. Mol. Biol. Plants.

[52] Zhan F., Li B., Jiang M., Yue X., He Y., Xia Y., Wang Y. (2018). Arbuscular mycorrhizal fungi enhance antioxidant defense in the leaves and the retention of heavy metals in the roots of maize. Environ. Sci. Pollut. Res..

[53] Zhou H.-Y., Nian F.-Z., Chen B.-D., Zhu Y.-G., Yue X.-R., Zhang N.-M., Xia Y.-S. (2023). Synergistic reduction of arsenic uptake and alleviation of leaf arsenic toxicity in maize (*Zea mays* L.) by arbuscular mycorrhizal fungi (AMF) and exogenous iron through antioxidant activity. J. Fungi.

[54] Ambawat S., Sharma P., Yadav N.R., Yadav R.C. (2013). MYB transcription factor genes as regulators for plant responses: An overview. Physiol. Mol. Biol. Plants.

[55] Cao Y., Li K., Li Y., Zhao X., Wang L. (2020). MYB Transcription Factors as Regulators of Secondary Metabolism in Plants. Biology.

[56] Zamioudis C., Hanson J., Pieterse C.M.J. (2014). β-Glucosidase BGLU42 is a MYB72-dependent key regulator of rhizobacteria-induced systemic resistance and modulates iron deficiency responses in *Arabidopsis* roots. New Phytol..

[57] Zhao H., Jiang J., Shen M., Zhang Y., Zhang Y., Liu H., Zhou H., Zheng Y. (2025). The transcription factor MYB30 promotes iron homeostasis by maintaining the stability of the FIT transcription factor. Plant Cell.

[58] Gong Q., Zhou M., Li X., Guo Y. (2024). Transcription factor MYB8 regulates iron deficiency stress response in *Arabidopsis*. Plant Sci..

[59] Meier R., Charvat I. (1993). Reassessment of tetrazolium bromide as a viability stain for spores of vesicular-arbuscular mycorrhizal fungi. Am. J. Bot..

[60] Hewitt E.J. (1966). Sand and water culture methods used in the study of plant nutrition. J. Assoc. Off. Anal. Chem..

[61] Phillips J.M., Hayman D.S. (1970). Improved procedures for clearing roots and staining parasitic and vesicular-arbuscular mycorrhizal fungi for rapid assessment of infection. Trans. Br. Mycol. Soc..

[62] Trouvelot A., Kough J., Gianinazzi-Pearson V., Gianinazzi-Pearson V., Gianinazzi S. (1986). Mesure du taux de mycorhization VA d’un système radiculaire: Recherche de méthode d’estimation ayant une signification fonctionnelle. Physiological and Genetical Aspects of Mycorrhizae.

[63] Zarcinas B.A., Cartwright B., Spouncer L.R. (1987). Nitric acid digestion and multi-element analysis of plant material by inductively coupled plasma spectrometry. Commun. Soil Sci. Plant Anal..

[64] Beauchamp C., Fridovich I. (1971). Superoxide dismutase: Improved assays and an assay applicable to acrylamide gels. Anal. Biochem..

[65] Chance B., Maehly A.C. (1955). Assay of catalases and peroxidases. Methods Enzymol..

[66] Beers R.F., Sizer I.W. (1952). A spectrophotometric method for measuring the breakdown of hydrogen peroxide by catalase. J. Biol. Chem..

[67] Heath R.L., Packer L. (1968). Photoperoxidation in isolated chloroplasts: I. Kinetics and stoichiometry of fatty acid peroxidation. Arch. Biochem. Biophys..

[68] Elstner E.F., Heupel A. (1976). Inhibition of nitrite formation from hydroxylammoniumchloride: A simple assay for superoxide dismutase. Anal. Biochem..

[69] Brennan T., Frenkel C. (1977). Involvement of hydrogen peroxide in the regulation of senescence in pear. Plant Physiol..

[70] Finn R.D., Bateman A., Clements J., Coggill P., Eberhardt R.Y., Eddy S.R., Heger A., Hetherington K., Holm L., Mistry J. (2014). Pfam: The protein families database. Nucleic Acids Res..

[71] Letunic I., Bork P. (2018). 20 years of the SMART protein domain annotation resource. Nucleic Acids Res..

