# Supplementary material for: Integrated Physiological and Transcriptomic Analyses Reveal That Arbuscular Mycorrhizal Symbiosis Enhances Iron Stress Tolerance in Eucalyptus grandis
Source: Plants (Basel). 2026 Jul 20;15(14):2213. doi: 10.3390/plants15142213 (PMC13417306; doi:10.3390/plants15142213)
Supplement: Supplementary file 1 [file plants-15-02213-s001.zip › plants-4415580-supplementary.pdf]

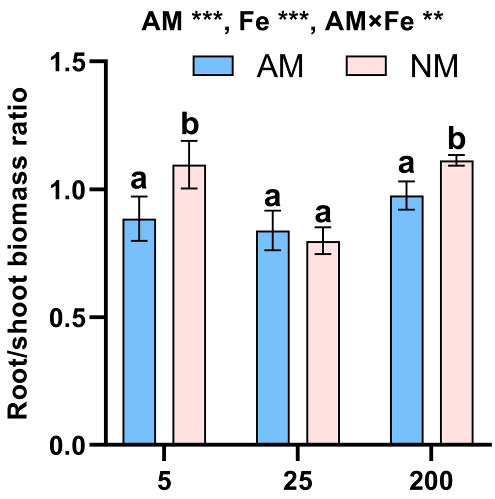


**Supplementary Figure S1. Root to shoot biomass ratio of *E. grandis* under different iron supply levels (5, 25, and 200 μM) with or without AM inoculation.** Different letters indicate significant differences (two-way ANOVA, Tukey's test, $P$<0.05). Data are means ± SE (n = 3). Significant effect: *: $P < 0.05$, **: $P < 0.01$, ***: $P < 0.001$, NS: no significant effect.

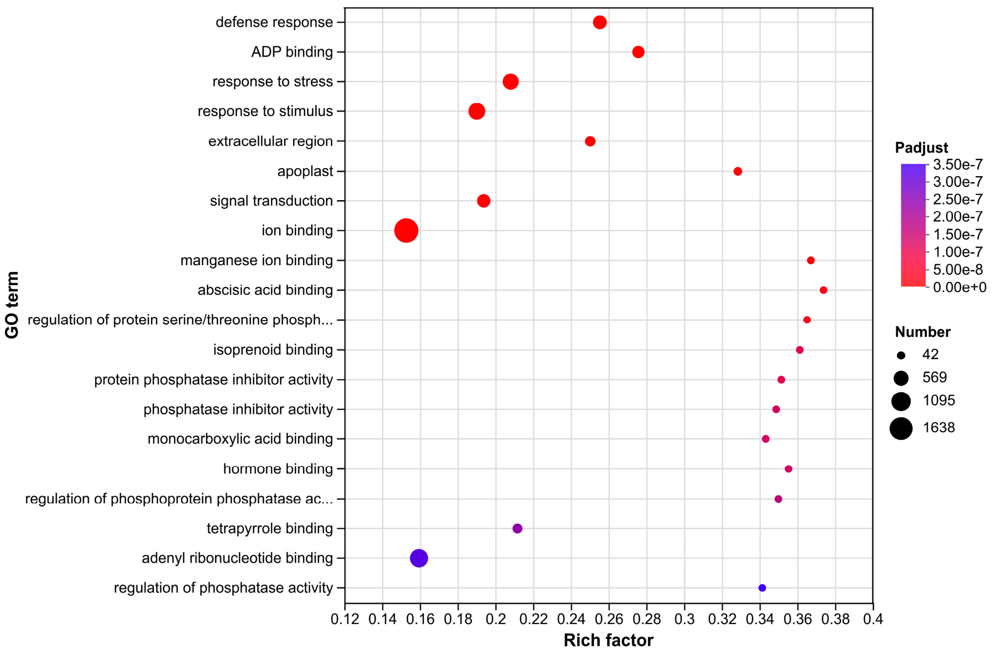


**Supplementary Figure S2. Gene Ontology (GO) enrichment analysis of differentially expressed genes (DEGs) in *E. grandis* roots under AM inoculation and iron stress.** The bubble plot shows the top 20 enriched GO terms (biological processes). The x-axis represents the Rich factor (the proportion of DEGs enriched in

a specific term relative to the total number of annotated genes in that term). The size of each bubble represents the number of DEGs; the color represents the adjusted P-value (red: lower P-value, indicating higher significance; blue: higher P-value). DEGs were identified with $|log_2FC| \geq 1$ and FDR < 0.05.

**Supplementary Table S1. Primer sequences used in this study**

| Name | Sequences (5'~3') | Purpose |
|---|---|---|
| EgMYB-2-F | GATCGAGGACAGAGAAGGTG | qRT-PCR |
| EgMYB-2-R | AGAGGATCGTTGAAGCTGTG | qRT-PCR |
| EgMYB-3-F | TCGATATCAAGAACAGCCTGC | qRT-PCR |
| EgMYB-3-R | CATCCTCAGTTGTCTCCCTTG | qRT-PCR |
| EgMYB315-1-F | ACCCATGTTTCGCAGAGTTC | qRT-PCR |
| EgMYB315-1-R | TTCTAAGTAATCAAGCCCATCCC | qRT-PCR |
| EgMYB315-2-F | ATCGCTTCCCATTTCCCTG | qRT-PCR |
| EgMYB315-2-R | TTCCTCTGTAATTGGCTGGTG | qRT-PCR |
| EgMYB61-F | AAGCTGAGGAAAGGTCTGTG | qRT-PCR |
| EgMYB61-R | ACCTTAACCGGCAACTCTTC | qRT-PCR |
| EgMYB306-F | AACAAGCCCTCTATGAAGCC | qRT-PCR |
| EgMYB306-R | TGCTATGTTCTTGGTGCTCG | qRT-PCR |
| EgUBI3-qF | TCACCTACGTCTACCAGAAGG | qRT-PCR |
| EgUBI3-qR | TCCTCGAAAGCTGTAAACATGG | qRT-PCR |
